# Cytokine-Mediated Regulation of ARG1 in Macrophages and Its Impact on the Control of *Salmonella enterica* Serovar Typhimurium Infection

**DOI:** 10.3390/cells10071823

**Published:** 2021-07-19

**Authors:** Natascha Brigo, Christa Pfeifhofer-Obermair, Piotr Tymoszuk, Egon Demetz, Sabine Engl, Marina Barros-Pinkelnig, Stefanie Dichtl, Christine Fischer, Lara Valente De Souza, Verena Petzer, Laura von Raffay, Richard Hilbe, Sylvia Berger, Markus Seifert, Ulrike Schleicher, Christian Bogdan, Günter Weiss

**Affiliations:** 1 Department of Internal Medicine II, Medical University of Innsbruck, Anichstraße 35, 6020 Innsbruck, Austria; natascha.brigo@i-med.ac.at (N.B.); christa.pfeifhofer@i-med.ac.at (C.P.-O.); piotr.s.tymoszuk@gmail.com (P.T.); egon.demetz@i-med.ac.at (E.D.); sabine.engl@tirol-kliniken.at (S.E.); marina.barros-pinkelnig@i-med.ac.at (M.B.-P.); sdichtl@biochem.mpg.de (S.D.); christine.fischer@i-med.ac.at (C.F.); lara.valente@i-med.ac.at (L.V.D.S.); verena.petzer@i-med.ac.at (V.P.); laura.von-raffay@i-med.ac.at (L.v.R.); richard.hilbe@tirol-kliniken.at (R.H.); sylvia.berger@i-med.ac.at (S.B.); markus.seifert@i-med.ac.at (M.S.); 2 Christian Doppler Laboratory for Iron Metabolism and Anemia Research, Medical University of Innsbruck, Anichstrasse 35, 6020 Innsbruck, Austria; 3 Mikrobiologisches Institut—Klinische Mikrobiologie, Immunologie, und Hygiene, Friedrich-Alexander-Universität (FAU) Erlangen-Nürnberg, Universitätsklinikum Erlangen, Wasserturmstraße 3/5, 91054 Erlangen, Germany; ulrike.schleicher@uk-erlangen.de (U.S.); christian.bogdan@uk-erlangen.de (C.B.); 4 Medical Immunology Campus Erlangen, FAU Erlangen-Nürnberg, 91054 Erlangen, Germany

**Keywords:** arginase 1, inducible nitric oxide synthase, *Salmonella enterica* serovar Typhimurium, macrophages, intracellular bacteria

## Abstract

Arginase 1 (ARG1) is a cytosolic enzyme that cleaves L-arginine, the substrate of inducible nitric oxide synthase (iNOS), and thereby impairs the control of various intracellular pathogens. Herein, we investigated the role of ARG1 during infection with *Salmonella enterica* serovar Typhimurium (*S*.tm). To study the impact of ARG1 on *Salmonella* infections in vitro, bone marrow-derived macrophages (BMDM) from C57BL/6N wild-type, ARG1-deficient Tie2Cre^+/−^ARG1^fl/fl^ and NRAMP^G169^ C57BL/6N mice were infected with *S*.tm. In wild-type BMDM, ARG1 was induced by *S*.tm and further upregulated by the addition of interleukin (IL)-4, whereas interferon-γ had an inhibitory effect. Deletion of ARG1 did not result in a reduction in bacterial numbers. In vivo, *Arg1* mRNA was upregulated in the spleen, but not in the liver of C57BL/6N mice following intraperitoneal *S*.tm infection. The genetic deletion of ARG1 (Tie2Cre^+/−^ARG1^fl/fl^) or its pharmacological inhibition with CB-1158 neither affected the numbers of *S*.tm in spleen, liver and blood nor the expression of host response genes such as iNOS, IL-6 or tumour necrosis factor (TNF). Furthermore, ARG1 was dispensable for pathogen control irrespective of the presence or absence of the phagolysosomal natural resistance-associated macrophage protein 1 (NRAMP1). Thus, unlike the detrimental function of ARG1 seen during infections with other intraphagosomal microorganisms, ARG1 did not support bacterial survival in systemic salmonellosis, indicating differential roles of arginine metabolism for host immune response and microbe persistence depending on the type of pathogen.

## 1. Introduction

Macrophages belong to the phagocytic cells of the innate immune system and represent one of the first lines of defence against invading pathogens. Depending on the type of activation, they are divided into two major forms: (I) classically activated or M1 macrophages that help to contain or clear microbes and express inducible or type 2 nitric oxide synthase (iNOS or NOS2); and (II) alternatively activated or M2 macrophages that support resolution of inflammation and tissue repair and express the enzyme arginase 1 (ARG1) [1,2,3,4,5,6].

The cytosolic enzyme ARG1 is upregulated by cytokines and microbial products during infections, which usually promotes pathogen survival in macrophages by converting L-arginine into urea and ornithine, thereby limiting the availability of L-arginine for iNOS to generate nitric oxide (NO) and L-citrulline. NO has been shown to exert central functions in host defence by acting as an anti-microbial agent but also as a regulator of immune effector functions. Interferon gamma (IFNγ) and tumour necrosis factor (TNF), along with bacterial lipopolysaccharides, are potent inducers of iNOS [7,8,9].

In addition, part of the host protective effects of TNF and IFNγ can be traced back to their inhibitory effect on the expression of ARG1, thereby promoting NO formation for antimicrobial host defence [10,11]. Anti-inflammatory cytokines such as interleukin (IL)-4 (IL-4) are potent inducers of ARG1. In contrast, TNF and IFNγ have been demonstrated to inhibit the transcription of ARG1 by hindering the IL-4-induced chromatin remodelling [10,11,12]. Specifically, in bone marrow-derived macrophages (BMDM) the simultaneous addition of TNF and IL-4 resulted in downregulation of *Arg1* mRNA and protein expression via TNF-mediated epigenetic inhibition of ARG1 induction by IL-4. As a consequence, in TNF-deficient mice the enzymatic activity of iNOS was reduced by the upregulation of ARG1, which was accompanied by a progression of *Leishmania* infection [10].

In the past, increased ARG1 activity has been linked to an impaired control of infections, especially with intracellular parasites [10,13,14,15,16]. The underlying mechanisms include reduction in L-arginine availability for high output formation of NO but also alterations of polyamine synthesis, or formation of antioxidants such as trypanothione, which can promote the growth of intracellular parasites such as *Trypanosoma* or *Leishmania* spp. The competition between iNOS and ARG1 for L-arginine has also been shown to be highly relevant for the control of infections with intracellular bacteria, such as *M. tuberculosis* [13,17], which are sensitive to NO-mediated killing.

While the function of ARG1 in infections with *Leishmania* spp. or mycobacteria is well established, little information is currently available on the role of ARG1 for the control of the Gram-negative bacterium *Salmonella enterica* serovar Typhimurium (*S*.tm). Similar to the other two pathogens, *S*.tm also resides in phagolysosomes and is susceptible to changes in micronutrient composition in this compartment as, for example, exerted by the transmembrane ion transporter NRAMP1 (natural resistance-associated macrophage protein 1; also termed solute carrier family 11 member A1, Slc11A1) [18,19]. NRAMP1 has been shown to be essential for resistance against infections with the intracellular microbes *S*.tm, *Leishmania donovani* and several mycobacterial species, including *Mycobacterium bovis* BCG [20]. In mice, one amino acid substitution, glycine to aspartic acid, leads to a non-functional NRAMP1 transporter [21]. Functional NRAMP1 transports iron, manganese and zinc ions. Previous studies showed that NRAMP1 functionality increased the expression of iNOS by activating the transcription factors IRF-1 and STAT-1. Due to high iNOS levels, NO production was enhanced and less intracellular pathogens were found in mice and macrophage cell lines expressing intact NRAMP1 [22,23,24,25].

Infections of mice with *S*.tm resemble human infections with *Salmonella* typhi, which can cause life-threatening systemic disease accounting for approximately 200,000 deaths per year worldwide. *S*.tm is phagocytosed by macrophages and able to evade their antimicrobial activities by inhibiting phagolysosomal fusion and impeding the recruitment of iNOS, thereby surviving and replicating inside the host [26,27,28,29,30]. Currently, treatment of invasive salmonellosis with antibiotics is often not successful, because resistance to conventional antibiotics is common. In addition, it is uncertain whether standard antibiotics are able to penetrate into the *Salmonella*-containing vacuole. Thus, it is necessary to better understand the host–pathogen interplay in salmonellosis and to identify new avenues for its treatment, either by strengthening anti-microbial host responses or by directly targeting the pathogen or its metabolism [31,32,33].

Here, we investigated the role of ARG1 in *Salmonella* infections in vitro and in vivo by analysing the antimicrobial activity of BMDM from C57BL/6N wild-type and ARG1-deficient Tie2Cre^+/–^ARG1^fl/fl^ mice and by monitoring the course of intraperitoneal *S*.tm infections in these mice as well as in wild-type littermates treated with an inhibitor of ARG1.

## 2. Materials and Methods

### 2.1. Isolation of Bone Marrow-Derived Macrophages and Culture Conditions

Tibiae and femurs from C57BL/6N, Tie2Cre^+/−^ARG1^fl/fl^, Tie2Cre^−/−^ARG1^fl/fl^ and NRAMP^G169^ C57BL/6N mice were used and flushed with cold phosphate-buffered saline (PBS; Lonza, Basel, Switzerland) supplemented with 1% penicillin/streptomycin (Sigma-Aldrich, St. Louis, MO, USA) as detailed elsewhere [34]. Cells were cultured for seven days in the presence of 50 ng/mL recombinant murine macrophage colony-stimulating factor (MCSF; Peprotech, Vienna, Austria) at 37 °C, 5% CO_2_ and saturated humidity. Medium was changed every second day. After differentiation of cells for six days, cells were washed with PBS and scraped. Cell numbers were determined with LUNA-FL fluorescent and bright field automated cell counter and by staining BMDM with acridine orange/propidium iodide (Biozym, Hessisch Oldendorf, Germany). BMDM were seeded into 6-well plates (Falcon^tm^, Szabo-Scandic, Vienna, Austria) at a density of 7 × 10^5^ cells per mL for in vitro infection experiments. For Western blot analyses, BMDM were seeded into petri dishes (diameter 10 cm; Falcon^tm^, Szabo-Scandic) after isolation.

### 2.2. In Vitro Infection

A pre-culture of *S*.tm (ATCC14028) in Lysogeny Broth (LB, ROTH, Karlsruhe, Germany) medium was shaken at 37 °C overnight. The following day, 50 μL of the bacterial suspension was transferred into 10 mL of LB medium and shaken for about 2 h at 37 °C in order to reach an optical density of 0.5 at 600 nm (OD600). This value was set to ensure that the bacteria were in the logarithmic growth phase. Viable *S*.tm were counted using the Casy counting system (OMNI Life Science, Bremen, Germany). Cells were infected for 1 h with a multiplicity of infection (MOI) of 10. Afterwards, *S*.tm that were not phagocytosed by BMDM were washed away with PBS + 25 mg/mL gentamicin (Gibco, Darmstadt, Germany), and 1 mL of DMEM media (10% FBS + 1% L-glutamine + 25 mg/mL gentamicin) was added to each well. Cells were either left unstimulated or stimulated with cytokines (10 ng/mL IL-4 (Peprotech) or 100 ng/mL IFNγ (Peprotech)) for various time points. To pharmacologically inhibit ARG1 in vitro, 50 µM of CB-1158 (MedchemExpress, Sollentuna, Sweden) dissolved in highly purified, sterile water for injection (Fresenius Kabi, Bad Homburg vor der Höhe, Germany) was added to each well for 14 and 24 h.

### 2.3. Animals

Mice were kept on a standard rodent diet (C2010 Altromin, Lage, Germany). The animals had free access to food and water and were kept according to institutional and governmental guidelines in the quarters of Medical University of Innsbruck with a 12 h dark-light cycle and an average temperature of 20 ± 1 °C. The animal experiments were approved by the Austrian Federal Ministry of Science and Research (BMWF-66.011/0139-V/3b/2018).

Tie2Cre^+/−^ARG1^fl/fl^ were generated by backcrossing of Tie2Cre-deleter mice and floxed ARG1 mice with C57BL/6N mice for 12 generations and inter-crossing thereafter. LoxP sites were inserted into the locus such that normal *Arg1* gene expression would not be disturbed except in Cre-expressing cells (endothelial cells, pericytes, subsets of hematopoietic progenitor cells and Tie2-expressing monocytes/macrophages) [13,35].

Breeding pairs of NRAMP^G169^ C57BL/6N mice were a kind gift from Ferric C. Fang (University of Washington, Seattle, WA, USA).

### 2.4. In Vivo Infection

In vivo infection with *S*.tm (ATCC 14028) was carried out as described [36]. Specifically, 500 colony-forming units (CFU) in 200 μL PBS were intraperitoneally (i.p.) injected into 8–12-week-old female mice (C57BL/6N, Tie2Cre^+/−^ARG1^fl/fl^ or Tie2Cre^−/−^ARG1^fl/fl^). After 72 h, mice were sacrificed and blood and organs were removed for further analysis. Complete blood count analysis was performed using a Vet-Animal Blood Counter (Scil Animal Care Co. GmbH, Viernheim, Germany).

### 2.5. Pharmacological Inhibition of ARG1 In Vivo

C57BL/6N mice were gavaged twice a day with 100 mg/kg of CB-1158 (MedchemExpress) dissolved in 200 µL highly purified, sterile water for injection (Fresenius Kabi) twice a day. CB-1158 is an ARG1 blocker with high oral bioavailability [37,38]. The control group was gavaged with water alone. After seven days mice were infected with 500 CFU of *S*.tm i.p. Treatment with 100 mg/kg of CB-1158 was continued until mice were sacrificed after 72 h of infection and blood and organs were taken for further analysis. Complete blood count analysis was performed using a Vet-Animal Blood Counter.

### 2.6. Colony Forming Units

The bacterial load in liver and spleen was determined by plating serial dilutions of homogenised infected organ pieces onto LB agar plates under sterile conditions. For in vitro experiments, cells were lysed with 1 mL of 0.5% sodium deoxycholic acid (Sigma-Aldrich). Serial dilutions of lysate were plated on LB agar plates. The colonies were counted manually after a 12 h incubation of the plates at 37 °C. For lysates of organs, the number of bacteria per gram of tissue was calculated and for cell culture and blood the CFU per mL was determined.

### 2.7. RNA Isolation, Reverse Transcription and TaqMan Quantitative Real-Time PCR (qRT-PCR)

Total RNA was extracted from cells and organs as described [34] using TRI Reagent^®^ (Sigma-Aldrich) according to the manufacturer’s protocol and reverse transcribed with M-MLV Reverse Transcriptase (Thermo Fisher Scientific, Waltham, MA, USA). The expression of genes listed in Table 1 was measured by quantitative real-time PCR using the CFX96 PCR system (BioRad, Feldkirchen, Germany) according to the instruction manual. The relative gene expression was calculated using the ∆∆Ct method.

In experiments with C57BL/6N BMDM, the mRNA expression of M1 and M2 macrophage markers (Table 2) was analysed with a QuantStudio Viia7 qPCR machine (Thermo Fisher Scientific) using gene-specific assays (TaqMan Gene Expression Assays, Thermo Fisher Scientific) and the TaqMan Universal Master Mix II, no UNG (Thermo Fisher Scientific). The relative gene expression was again calculated using the ∆∆Ct method.

### 2.8. Immunoblotting

Protein extracts from frozen mouse tissue or cells were prepared and immunoblotting was performed as described [39]. Staining with anti-ARG1, anti-iNOS and anti-β-ACTIN primary antibodies (Table 3) was detected with the ECL™ Prime Western Blotting System (Amersham Biosciences Europe GmbH, Freiburg, Germany) using a ChemiDoc Imaging system (Biorad).

### 2.9. Flow Cytometry

Splenocytes collected from the spleens of infected and uninfected mice were stained with monoclonal antibodies directed against cell surface markers as described [40]. Afterwards, cells were fixed and permeabilised with BD Cytofix/Cytoperm™ (BD Biosciences, Heidelberg, Germany) solution and stained with an antibody against iNOS. As controls, single marker stainings and FMO (fluorescent minus one) stainings were performed. The analysis was carried out with a Beckman–Coulter CytoflexS device and analysed with FlowJo software (10.6.1, FlowJo LLC, Ashland, OR, USA). The fluorochrome-conjugated antibodies used are listed in Table 4.

### 2.10. Detection of Interleukins and Nitrite

Detection and concentrations of TNF (R&D, Minneapolis, MN, USA) and IL-6 (BD Biosciences) in plasma was determined by ELISA following the manufacturer’s protocol. The concentration of nitrite, a stable oxidation product of NO, was determined with the Griess–Ilosvay’s nitrite reagent (Merck, Darmstadt, Germany) as previously described [41].

### 2.11. Urea Assay

The concentration of urea in the plasma of infected mice was assessed using the Urea Assay Kit (Abcam, ab83362, Cambridge, UK) following the manufacturer’s protocol. Briefly, 0.1–1 µL of sample was mixed with the provided reaction mix in a 96-well plate. The plate was incubated at 37 °C for 1 h. Thereafter, OD was measured at 570 nm with a Tecan microplate reader. The concentration of urea was calculated based on a urea standard curve.

### 2.12. Arginase 1 Activity Assay

Enzymatic arginase 1 activity was measured as previously described [42]. Briefly, cell lysates were prepared [39] and activated at 56 °C after adding 25 mM Tris pH 7.4 (Roth) buffer with manganese (II) chloride (MnCl_2_; Sigma-Aldrich) with a final concentration of 2 mM MnCl_2_. Thereafter, 0.5 M L-arginine (Sigma-Aldrich) was added at a pH of 9.7 and a temperature of 37 °C. The reaction was stopped by adding an acid mixture of H_2_SO_4_ (96%; Roth), H_3_PO_4_ (85%; Roth) and H_2_O in a proportion of 1:3:7. The stable end product, urea, was detected by adding α-isonitrosopropiophenone (ISPF; Sigma-Aldrich) for 1 h at 95 °C, which led to a colour change. The absorption was measured at 540 nm using a SPECTRA max microplate reader (Molecular Devices, San Jose, CA, USA).

### 2.13. Statistical Analysis

Statistical differences were analysed with Graph Pad Prism 8. The data in the graphs are presented as means ±  SEM. In general, results are shown as bars and whisker plots with each symbol representing an animal or individual analysis. Two groups were analysed for their significant differences by a two-tailed Student’s *t*-test. For more groups/factors, one or two-way ANOVA with a Dunnett post hoc test was used. *p*-values < 0.05 were considered significant.

## 3. Results

### 3.1. IL-4-Dependent Arg1 Upregulation Is Ameliorated upon S.tm Infection

**Figure 1:**
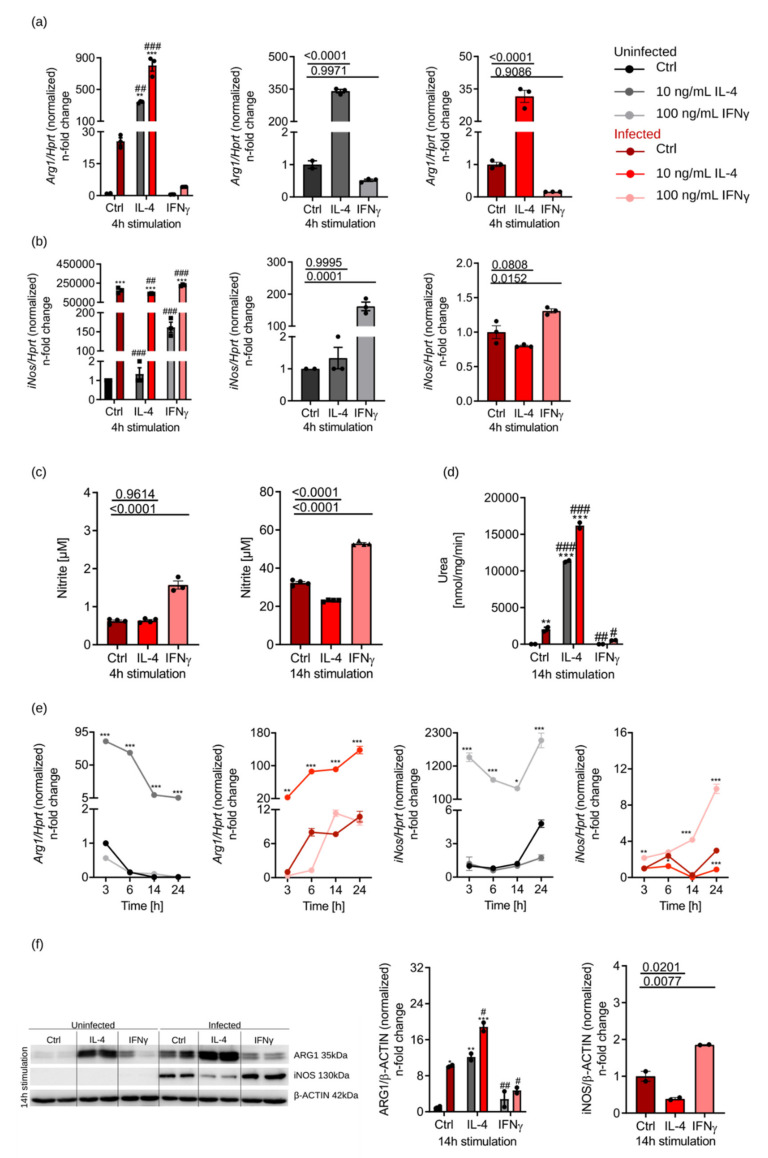
ARG1 is upregulated by IL-4 and further enhanced after *S*.tm infection, but blocked by IFNγ. BMDM of C57BL/6N mice were infected (different shades of red) with *S*.tm and stimulated with IL-4 (10 ng/mL) or IFNγ (100 ng/mL) for 3–24 h. Uninfected (different shades of grey) and unstimulated BMDM (Ctrl) were used as a control. (**a**,**b**) Regulation of *Arg1* and *iNos* expression due to infection and stimulation determined by RT-qPCR analysis. mRNA levels were normalised to the expression of the control gene hypoxanthin-guanin-phosphoribosyltransferase (*Hprt*). Ctrl samples were set to 1. Significant differences as determined by two-way ANOVA are marked **;## = *p*-value < 0.01; ***;### = *p*-value < 0.001. * indicates significant differences to uninfected Ctrl; # depicts significant differences to infected Ctrl. Otherwise, three groups were statistically analysed by one-way ANOVA. Exact *p*-values are indicated. (**c**) Nitrite concentrations in culture supernatants of infected BMDM as determined by the Griess reaction. Groups were statistically analysed by one-way ANOVA. Exact *p*-values are indicated. (**d**) ARG1 enzyme activity determined by measuring urea concentrations in cell lysates. Groups were statistically analysed by two-way ANOVA. Exact *p*-values are marked # = *p*-value < 0.05; **;## = *p*-value <0.01; ***;### = *p*-value < 0.001. * indicates significant differences to uninfected Ctrl; # depicts significant differences to infected Ctrl. (**e**) Cytokine-dependent regulation of *Arg1* and *iNos* in uninfected and infected BMDM at different time points of stimulation (3–24 h). mRNA levels were normalized to the expression of the control gene *Hprt* and the 3 h Ctrl group was set to 1. Significant differences as determined by two-way ANOVA are marked * = *p*-value < 0.05; ** = *p*-value < 0.01; ***= *p*-value < 0.001. * indicates significant differences to uninfected Ctrl. (**f**) Protein expression of ARG1 and iNOS and densitometric quantification of results relative to β-ACTIN expression. Groups were statistically analysed by two-way ANOVA. Exact *p*-values are marked *;# = *p*-value < 0.05; **;## = *p*-value < 0.01; *** = *p*-value < 0.001. * indicates significant differences to uninfected Ctrl; # depicts significant differences to infected Ctrl. Otherwise, three groups were statistically analysed by one-way ANOVA. Exact p-values are indicated. Representative data of technical triplicates or quadruplicates are shown for (**a**–**c**) and (**e**); technical duplicates are shown for (**d**) and (**f**).

To study the regulation of ARG1 by IL-4 and IFNγ, we stimulated BMDM of C57BL/6N mice with 10 ng/mL IL-4 or 100 ng/mL IFNγ for various time points. Furthermore, we investigated whether *Arg1* regulation differs between infected and uninfected samples (Figure 1a). In uninfected macrophages, we found that *Arg1* expression was increased by IL-4 and reduced by IFNγ. Infection with *S*.tm induced *Arg1* expression and IL-4 had an additive effect, whereas IFNγ reduced *Arg1* mRNA expression (Figure 1a). *iNos* mRNA levels were upregulated by IFNγ and further enhanced by *S*.tm infection, whereas IL-4 had only insignificant effects on *iNos* mRNA expression (Figure 1b). To study whether IL-4 stimulation also affected the expression of other genes involved in macrophage polarisation, we quantified the expression of M2-like phenotype markers chitinase-like 3 (*Chil3*) and mannose receptor C-type 1 (*Mrc1*) (Supplemental Figure S1a), which were upregulated to a comparable extent as *Arg1*. On the other hand, M1-macrophage-specific markers (Figure S1b), such as *Tnf*, *Il-6* and *iNos,* were induced by IFNγ. Of note, M1 and M2 markers were generally further increased by co-infection with *S*.tm. Moreover, we analysed the effects of pro- and anti-inflammatory cytokines on iNOS activity by measuring nitrite in culture supernatants of BMDM (Figure 1c). We confirmed that IFNγ increased nitrite formation in *Salmonella*-infected macrophages, whereas IL-4 reduced the generation of NO over time. In addition, ARG1 enzyme activity was determined (Figure 1d). In uninfected samples, urea was only detectable in IL-4-stimulated samples. Infection with *S*.tm led to measurable urea levels under all stimulation conditions. Addition of IL-4 further increased the urea levels compared to control samples. IFNγ treatment resulted in lower urea concentrations, nicely resembling the effects of cytokines and infection on *Arg1* expression.

Having seen this principal regulation of *iNos* and *Arg1* by cytokines and *Salmonella,* we studied their expression over time (3–24 h) (Figure 1e). *iNos* mRNA levels were strongly induced by IFNγ in both uninfected and infected macrophages (Figure 1e), which, however, did not translate into induction of iNOS protein in uninfected macrophages stimulated with IFNγ (Figure 1f). Only in infected macrophages, IFNγ resulted in detectable iNOS protein levels and increased iNOS activity (Figure 1c,f). This indicated that *S*.tm infection of BMDM is a strong inducer of iNOS protein. Unlike IFNγ, IL-4 reduced the expression of iNOS protein and the production of NO (Figure 1c,f), which is in agreement with the observed roles of these cytokines in NO formation [9].

With regard to *Arg1* mRNA and protein expression, this was rapidly induced by IL-4, but then declined in uninfected BMDM over time, whereas a continuous increase in and super induction of *Arg1* expression was observed in infected cells (Figure 1e). On the protein level, infection with *S*.tm alone already caused induction of ARG1 protein, which was further increased in the presence of IL-4, but suppressed by IFNγ (Figure 1f). After 24 h of infection and IL-4 stimulation, we detected the highest levels of ARG1 and iNOS proteins (details not shown).

From these data, we conclude that *S*.tm cooperates with IL-4 for the induction of ARG1 in BMDM and leads to higher ARG1 levels over time.

### 3.2. ARG1-Deficient BMDM from Tie2Cre^+/−^ARG1^fl/fl^ Mice Did Not Show an Improved Control of S.tm Infection

**Figure 2:**
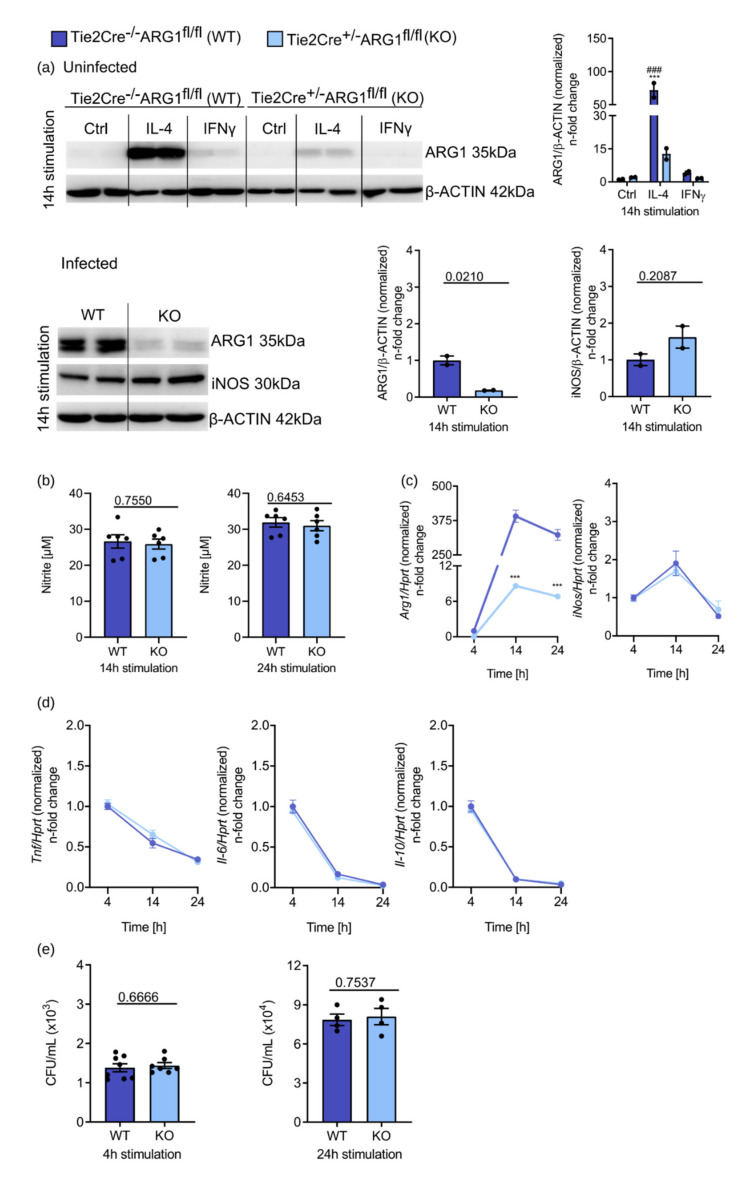
Deletion of ARG1 does not lead to improved infection control in macrophages. BMDM of Tie2Cre^+/−^ARG1^fl/fl^ knockout mice (light blue) and Tie2Cre^−/−^ARG1^fl/fl^ wild-type littermates (dark blue) were stimulated with IL-4 (10 ng/mL) or IFNγ (100 ng/mL) for 14 h. Unstimulated BMDM were used as a control. In addition, they were infected with *S*.tm for 4, 14 and 24 h. (**a**) Protein levels of ARG1 and iNOS and densitometric quantification of results relative to β-ACTIN expression. Significant differences as determined by two-way ANOVA are marked ***;### = *p*-value < 0.001. * indicates significant differences to uninfected Ctrl of Tie2Cre^−/−^ARG1^fl/fl^ BMDM; # depicts significant differences to uninfected Ctrl of Tie2Cre^+/−^ARG1^fl/fl^ BMDM. Otherwise, two groups were statistically analysed by Student’s *t*-test. Exact *p*-values are indicated. (**b**) Nitrite concentrations in culture supernatants of infected BMDM determined by the Griess reaction. Significant differences as determined by Student’s *t*-test are shown. Exact *p*-values are indicated. (**c**) Regulation of *Arg1* and *iNos* expression during infection determined by RT-qPCR analysis. mRNA levels were normalised to the control *Hprt*. Tie2Cre^−/−^ARG1^fl/fl^ samples 4 h after infection were set to 1. Significant differences as determined by two-way ANOVA. *p*-values are indicated (*** ≤ 0.001). * indicates significant differences to Tie2Cre^−/−^ARG1^fl/fl^ sample 4 h after infection. (**d**) RT-qPCR analysis of inflammatory markers (*Il-6*, *Tnf* and *Il-10*). mRNA levels were normalised to the control *Hprt*. Tie2Cre^−/−^ARG1^fl/fl^ samples 4 h after infection are set to 1. Significant differences were determined by two-way ANOVA. (**e**) Determination of viable *S*.tm in macrophages after 4 and 24 h of infection. Significant differences as determined by Student’s *t*-test. Exact *p*-values are indicated. Representative data from three independent experiments performed with duplicates or triplicates are shown.

To study the impact of ARG1 on the course of *S*.tm infection, we isolated BMDM from Tie2Cre^+/−^ARG1^fl/fl^ (deletion of ARG1 in endothelial cells, pericytes, subsets of hematopoietic stem cells and Tie2-expressing monocytes/macrophages [13,35]) and Tie2Cre^−/−^ARG1^fl/fl^ wild-type littermates [10,13,14,15,16]. Isolated BMDM were either left unstimulated or treated with 10 ng/mL IL-4 or 100 ng/mL IFNγ for 14 h or infected with *S*.tm. Protein levels of ARG1 and iNOS were compared between conditional ARG1 knockout mice and wild-type littermates (Figure 2a). Uninfected BMDM from Tie2Cre^−/−^ARG1^fl/fl^ control mice showed the same protein up- or downregulation of ARG1 as observed for BMDMs isolated from C57BL/6N mice (see Figure 1f above). IL-4 led to a strong increase in ARG1 protein in Tie2Cre^−/−^ARG1^fl/fl^ BMDM, which was only weakly seen in BMDM from Tie2Cre^+/−^ARG1^fl/f^ (the residual ARG1 protein expression is likely due to incomplete gene deletion) (Figure 2a). Of interest, upon *S*.tm infection ARG1 was strongly expressed in wild-type BMDM even in the absence of IL-4, but not in Tie2Cre^+/−^ARG^fl/fl^ BMDM (Figure 2a). Additionally, deletion of ARG1 was associated with an increase in iNOS protein expression in those samples (Figure 2a), which, however, did not translate into increased nitrite formation (Figure 2b). The efficient suppression of *Arg1* mRNA levels in infected Tie2Cre^+/−^ARG1^fl/fl^ BMDM was also seen over a period of 24 h (Figure 2c). In contrast, analysis of *iNos* mRNA expression yielded comparable results in infected Tie2Cre^−/−^ARG1^fl/fl^ and Tie2Cre^+/−^ARG1^fl/fl^ BMDM (Figure 2c), which also held true when studying the expression of important cytokines involved in anti-microbial host defence such as *Tnf*, *Il-6* or *Il-10* (Figure 2d). *iNos* mRNA levels were highest 14 h after infection and decreased thereafter, whereas *Tnf*, *Il-6* and *Il-10* decreased continuously over time. Finally, when we examined the effects of ARG1 on bacterial numbers in infected BMDM, no differences between CFU counts in the two groups were seen at 4 and 24 h after infection (Figure 2e).

Thus, the deletion of ARG1 in macrophages neither resulted in a better infection control of intracellular *Salmonella* nor in an altered expression of important host immune response genes in vitro.

### 3.3. ARG1-Deficient Tie2Cre^+/−^ARG1^fl/fl^ Mice Showed Similar S.tm Control In Vivo as Compared to Wild-Type Littermates

**Figure 3:**
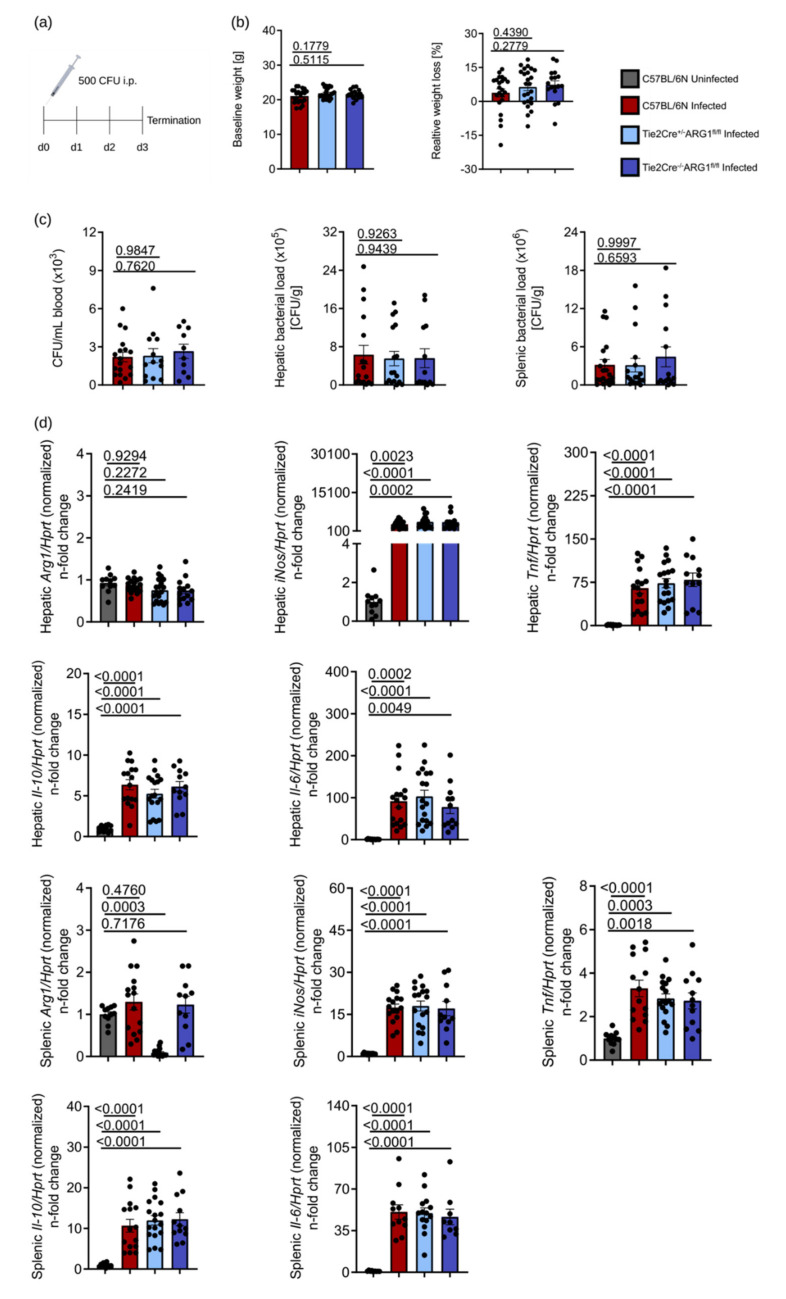

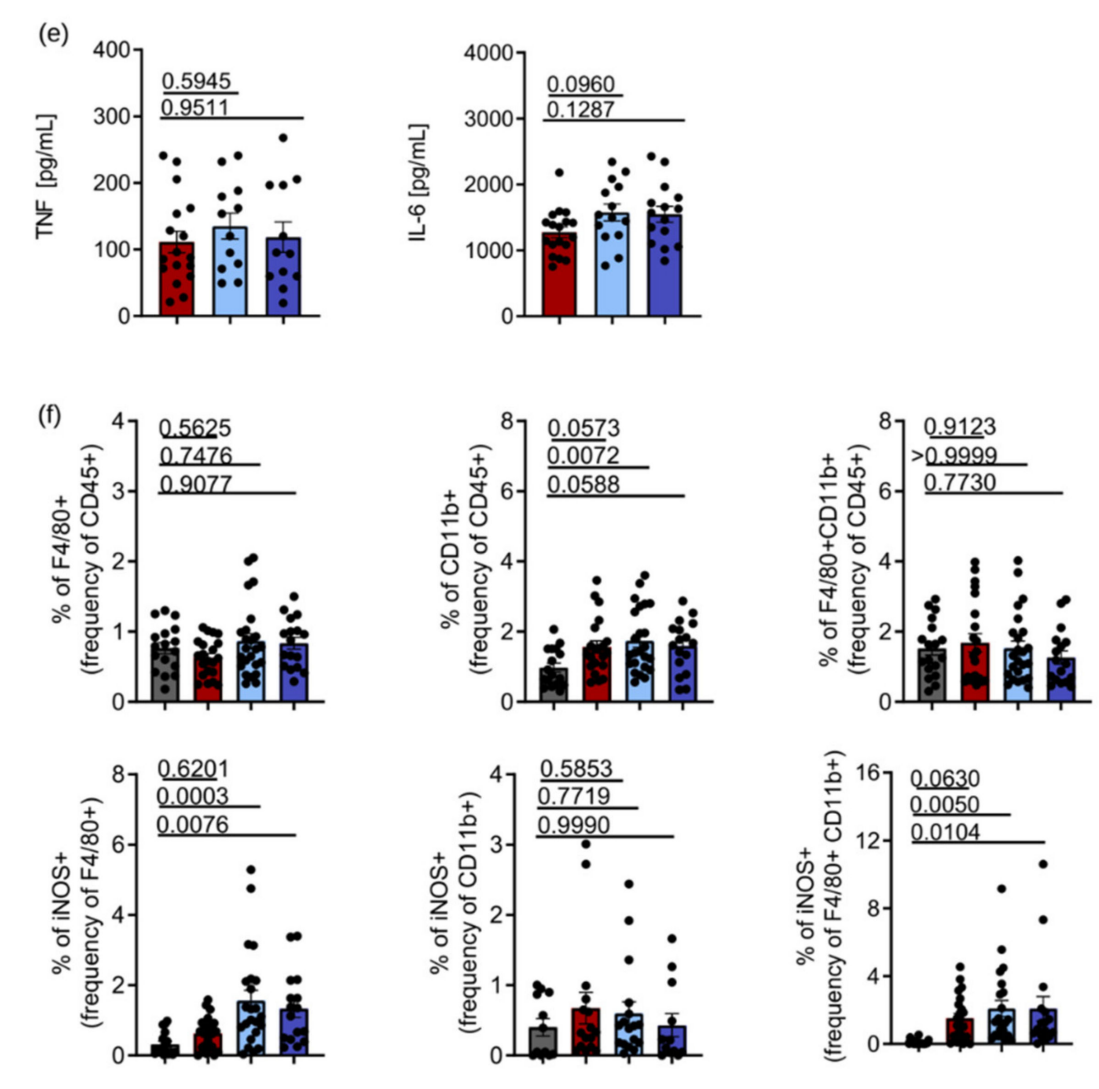
Tie2Cre^+/−^ARG1^fl/fl^ knockout mice showed the same course of infection as Tie2Cre^−/−^ARG1^fl/fl^ littermates and C57BL/6N mice. Tie2Cre^+/^^−^ARG1^fl/fl^ knockout mice (light blue), Tie2Cre^−^^/−^ARG1^fl/fl^ littermates (dark blue) and C57BL/6N (red) were infected with 500 CFU of *S*.tm for 72 h. Uninfected mice (grey) were used as controls. After 72 h, mice were sacrificed and blood and organs harvested. (**a**) Schematic representation of the *S*.tm infection experiment. (**b**) Initial weight of mice and relative weight loss at termination of the experiment. (**c**) Bacterial load in blood (CFU/mL), liver and spleen (CFU/gram tissue) of infected mice. (**d**) RT-qPCR analysis of *Arg1*, *iNos*, *Tnf*, *Il-6* and *Il-10* in liver and spleen samples. mRNA levels were normalised to the control *Hprt*. Uninfected samples were set to 1. (**e**) Plasma TNF and IL-6 levels determined by ELISA. (**f**) Spleen cell suspensions were analysed for the expression of surface markers (F4/80 and CD11b) and iNOS by flow cytometry. Representative data from four independent experiments performed with 5–8 mice per group are shown. Graphs show mean ± SEM. Significant differences were determined by one-way ANOVA. Exact *p*-values are indicated.

Although our cell culture results did not suggest a role for ARG1 in the control of *S*.tm infection in BMDM, we considered the possibility that the chosen in vitro conditions might obviate the detection of an impact of ARG1. We therefore investigated whether an ARG1 deletion has an effect on the course of infection with *S*.tm in vivo. We injected C57BL/6N, Tie2Cre^+/−^ARG1^fl/fl^ and Tie2Cre^−/−^ARG1^fl/fl^ mice with 500 CFU of *S*.tm intraperitoneally (i.p.) (Figure 3a). The visceral infection of mice with *S*.tm caused comparable weight loss in all three mouse lines when related to the respective baseline values (Figure 3b). After 72 h of infection, mice were sacrificed and bacterial counts were determined in the blood and in organs (Figure 3c). ARG1-deficient Tie2Cre^+/−^ARG1^fl/fl^ mice showed no reduction in CFU levels in blood, liver and spleen when compared to the two other mouse strains. When studying *Arg1* mRNA expression in the liver, we neither observed an upregulation following infection nor differences between the various genotypes, which is likely due to the high constitutive expression of *Arg1* in hepatocytes. In contrast, *Arg1* mRNA expression was significantly lower in the spleen of Tie2Cre^+/−^ARG1^fl/fl^ mice as compared to the two other groups, suggesting that macrophages and/or endothelial cells prominently contribute to the *Arg1* expression in the spleen. As observed in vitro, the expression of all macrophage immune response genes analysed (*Tnf*, *Il-6*, *Il-10* and *iNos*) was markedly increased upon infection with *S*.tm but no differences were noted between the various genotypes after 72 h of infection (Figure 3d). Additionally, we determined the levels of IL-6 and TNF in plasma of infected mice, which revealed no differences between ARG1-proficient and ARG1-deficient mice, confirming our tissue expression analysis (Figure 3e). Since *S*.tm are phagocytosed by macrophages, we analysed splenic tissue lysates for differences in the composition of various macrophage populations by FACS (Figure 3f). The gating strategy of the ex vivo flow cytometric staining is shown in Supplemental Figure S2. Neither the relative numbers of F4/80^+^, CD11b^+^ and F4/80^+^/CD11b+ cells nor the relative numbers of iNOS-expressing macrophages were different between the three groups following infection (Figure 3f).

Together with our in vitro data, these in vivo results demonstrate that deletion of ARG1 in Tie2Cre-expressing cells does not cause any reduction in the *S*.tm burden in blood, liver and spleen.

### 3.4. Pharmacological Inhibition of ARG1 with CB-1158 Did Not Affect the Control of Systemic Salmonella Infection in Mice

**Figure 4:**
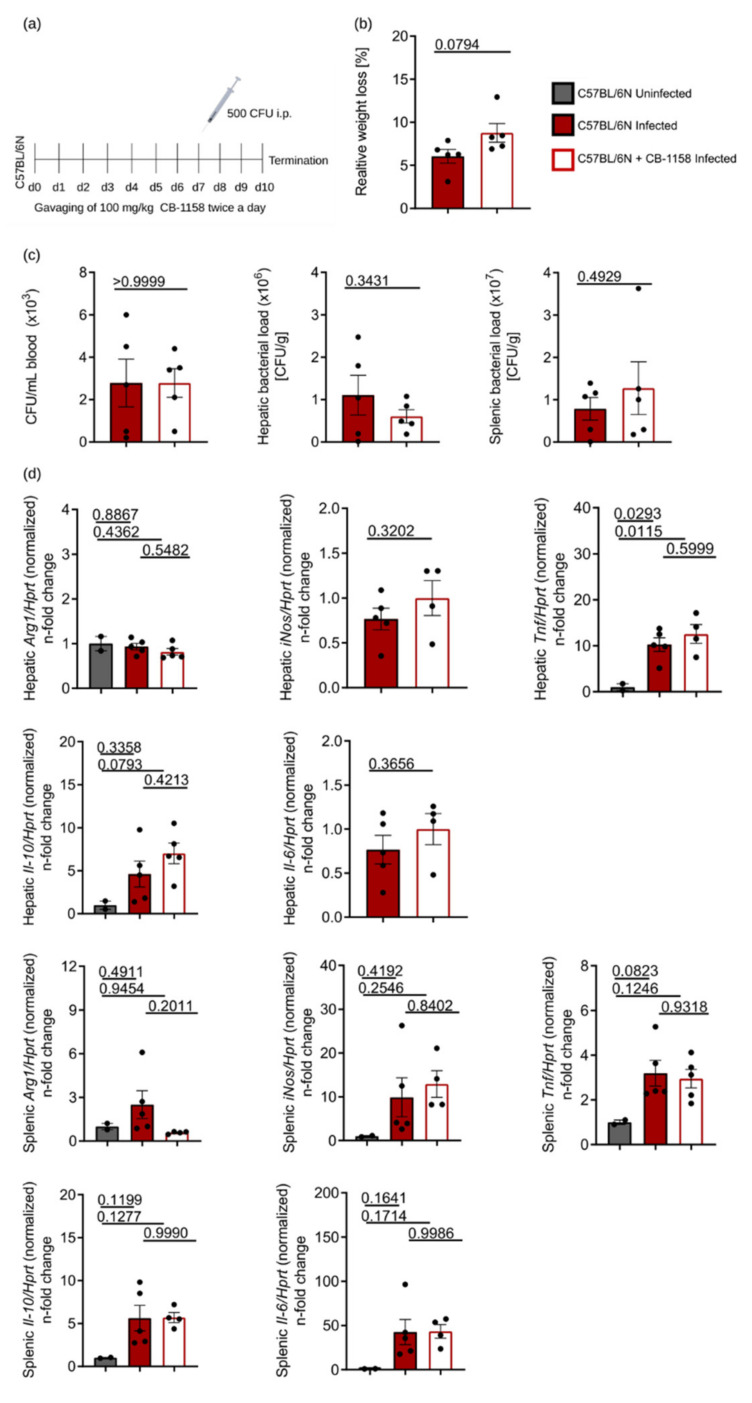

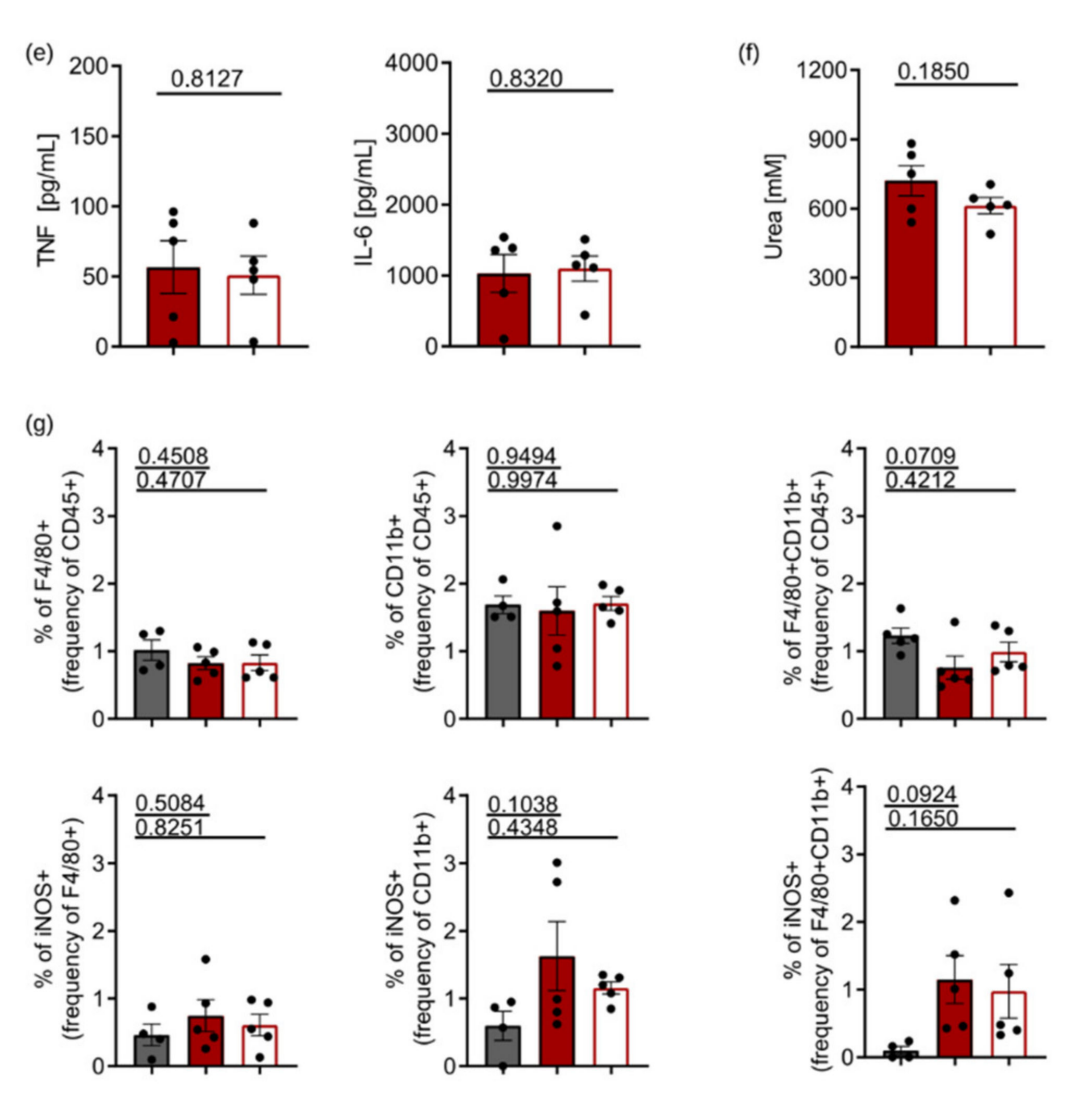
Mice treated with the ARG1 inhibitor CB-1158 showed a similar infection profile as infected, but untreated C57BL/6N control mice. C57BL/6N mice were either treated with 100 mg/kg CB-1158 twice a day or treated with solvent alone. Treatment started at day 7 prior to infection and was continued for 72 h following infection with 500 CFU of *S*.tm. Uninfected mice were used as controls. After 72 h mice were sacrificed and blood and organs were harvested. (**a**) Schematic representation of the treatment and infection protocol. (**b**) Relative weight loss at termination of the experiment. (**c**) Bacterial load in blood (CFU/mL), liver and spleen (CFU/gram tissue) of infected mice. (**d**) RT-qPCR analysis of *Arg1*, *iNos*, *Tnf*, *Il-6* and *Il-10* in liver and spleen samples. mRNA levels were normalised to the control *Hprt*. Uninfected mice were set to 1. (**e**) Plasma TNF and IL-6 levels determined by ELISA. (**f**) Determination of arginase enzyme activity by measurement of urea in plasma. (**g**) Spleen cell suspensions were analysed for surface markers (F4/80 and CD11b) and iNOS by flow cytometry. Data obtained with 5 mice per group are shown and represent mean ± SEM. Significant differences were determined by Student’s *t*-test (two groups) and one-way ANOVA (three groups). Exact *p*-values are indicated.

It is possible that the deletion of ARG1 in Tie2Cre-expressing cells will be compensated by ARG1 expression in other cell types. As mice with a universal deletion of ARG1 are not viable [35,43], we performed additional infection experiments in which we applied the pharmacological ARG1 inhibitor CB-1158. To this end, we gavaged C57BL/6N wild-type mice twice a day with CB-1158 for 7 days prior to infection and throughout the subsequent course of infection over 72 h (Figure 4a). CB-1158-treated mice lost minimally more weight during the course of infection than their infected littermates that were gavaged with the solvent only (Figure 4b). However, treatment with CB-1158 did not result in an altered control of infection as reflected by unchanged CFU counts in blood, liver and spleen samples (Figure 4c). Of note, *Arg1* mRNA expression in the spleen was lower in infected and CB-1158-treated mice as compared to infected and untreated littermates (Figure 4d), whereas *Arg1* expression in the liver was neither induced by infection nor reduced by CB-1158, largely confirming the results obtained with ARG1-deficient Tie2Cre^+/−^ARG1^fl/fl^ mice (Figure 3d). In addition, we did not find any differences in the mRNA expression or circulating plasma levels of *iNos*, *Tnf*, *Il-6* or *Il-10* when comparing infected mice with their littermates receiving CB-1158 (Figure 4d,e). To test whether the activity of ARG1 was reduced by CB-1158, we measured urea in the plasma of infected and CB-1158-treated mice (Figure 4f). We found a slight although insignificant reduction in urea in the CB-1158-treated mice, which might be due to the fact that CB-1158 poorly blocks hepatic ARG1 and thereby does not cause a strong reduction in urea plasma levels [37]. Furthermore, we investigated macrophage subpopulations and the number of iNOS-expressing cells by flow cytometry, which again did not reveal any differences in the relative numbers of F4/80^+^, CD11b^+^ or F4/80^+^/CD11b^+^ double-positive cells or in the percentage of iNOS-expressing cells in *S*.tm-infected mice (Figure 4g). The gating strategy of the ex vivo flow cytometric staining is shown in Figure S2.

### 3.5. ARG1 Is Dispensable for the Control of Salmonella by Macrophages Irrespective of the Presence or Absence of Functional NRAMP1

**Figure 5:**
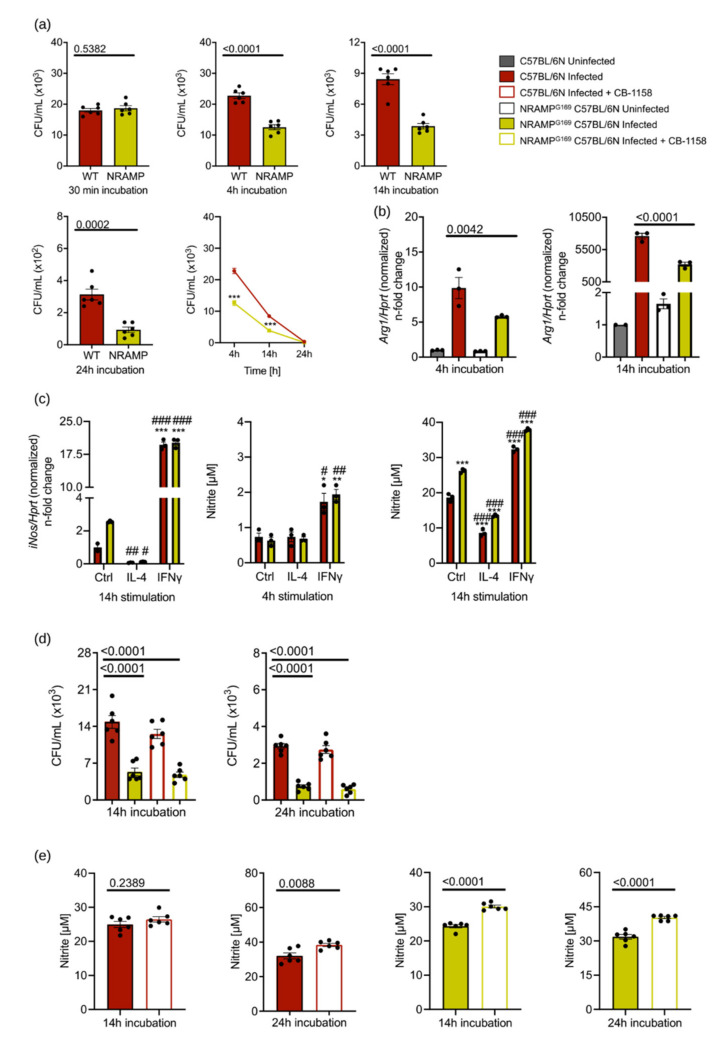
NRAMP1 expression has no impact on the role of ARG1 in the control of *S*.tm infection. BMDM of NRAMP^G169^ C57BL/6N mice expressing functional NRAMP and BMDM from C57BL/6N were infected with *S*.tm and either left unstimulated or stimulated with 10 ng/mL IL-4 or 100 ng/mL IFNγ for 4–24 h. Furthermore, infected BMDM were stimulated with 50 µM CB-1158 for 14 and 24 h. (**a**) Bacterial burden at 30 min after infection and survival of *S*.tm in BMDM after 4, 14 and 24 h. Groups were statistically analysed by Student’s *t*-test. Exact *p*-values are indicated. CFU time course was statistically analysed by two-way ANOVA (*** = *p*-value < 0.001). * indicates significant differences to infected BMDM from C57BL/6N at specific time point. (**b**) Regulation of *Arg1* expression upon infection and cytokine stimulation as determined by RT-qPCR analysis after 4 and 14 h. mRNA levels were normalised to the control *Hprt*. Uninfected Ctrl samples of C57BL/6N BMDM were set to 1. Significant differences were determined by one-way ANOVA. Exact *p*-values are indicated. (**c**) *iNos* expression after infection and cytokine stimulation as determined by RT-qPCR analysis after 14 h. mRNA levels were normalised to the control *Hprt*. Infected Ctrl samples of C57BL/6N BMDM were set to 1. Corresponding nitrite concentrations in culture supernatants of infected BMDM were determined by the Griess reaction. Groups were statistically analysed by two-way ANOVA. *p*-values are marked *;# = *p*-value < 0.05; **;## = *p*-value < 0.01; ***;### = *p*-value < 0.001. * indicates significant differences to infected unstimulated BMDM from C57BL/6N; # depicts significant differences to infected unstimulated BMDM of NRAMP^G169^ C57BL/6N. (**d**) Bacterial numbers in BMDM after 14 and 24 h of infection in the presence/absence of 50 µM CB-1158. Groups were statistically analysed by one-way ANOVA. Exact *p*-values are indicated (**e**) Nitrite concentrations in culture supernatants of infected versus infected and CB-1158-treated BMDM as determined by the Griess reaction. Groups were statistically analysed by Student’s *t*-test. Exact *p*-values are indicated. Representative data of triplicates of two experiments (**a**,**d**,**e**) or technical triplicates (**b**,**c**) are shown. Graphs show mean ± SEM.

A functional NRAMP1 (Slc11A1) transporter has been described to be important for the control of infections with intracellular pathogens, including *S*.tm in mice. To exclude the possibility that the unaltered course of *S*.tm infection observed in ARG1-deficient mice is causally linked to a non-functional NRAMP1 in C57BL/6N mice, we performed a comparative analysis of BMDM obtained from C57BL/6N mice (expressing non-functional NRAMP1) and NRAMP^G169^ C57BL/6N mice (expressing functional NRAMP1). Bacterial numbers were identical 30 min after infection in macrophages of both genotypes. After 4, 14 and 24 h, we found that BMDM of NRAMP^G169^ C57BL/6N mice killed significantly more *S*.tm than BMDM from C57BL/6N (Figure 5a). Both at 4 and 14 h after infection, *Arg1* expression was higher in infected BMDM of C57BL/6N than in infected BMDM of NRAMP^G169^ C57BL/6N mice (Figure 5b). As seen before (Figure 1a), infection with *S*.tm caused an upregulation of *Arg1* in BMDM of both genotypes (Figure 5b). As previously shown [22,23,24,25], NRAMP^G169^ C57BL/6N BMDM showed higher *iNos* levels in infected samples. Stimulation of NRAMP1-expressing BMDM with IL-4 and IFNγ led to the same pattern of regulation of *Arg1* as seen in Figure 1b. Thus, IL-4 reduced *iNos* expression whereas IFNγ elevated its levels. Furthermore, nitrite levels correlated with *iNos* expression levels after 14 h. At 14 h after infection, unstimulated, IL-4- or IFNγ-treated NRAMP^G169^ C57BL/6N BMDM showed significantly higher nitrite levels compared to C57BL/6N BMDM (Figure 5c). As *Arg1* expression was different between infected macrophages of both genotypes, we then investigated the effect of pharmacological inhibition of ARG1 with CB-1158 on the fate of *Salmonella* in macrophages depending on the NRAMP1 genotype. While NRAMP^G169^ C57BL/6N BMDM contained lower numbers of intracellular *Salmonella* at 14 and 24 h after infection than C57BL/6N BMDM (Figure 5d), blockade of ARG1 activity did not change the bacterial load. Of note, ARG1 inhibition resulted in slightly increased nitrite levels in infected BMDM expressing functional NRAMP1 (Figure 5e). Taken together, these data demonstrate that inhibition of ARG1 by CB-1158 did not reduce the bacterial burden in macrophages, irrespective of the presence or absence of functional NRAMP1.

## 4. Discussion

The different L-arginine catabolizing pathways have been shown to be of central importance for host resistance to infection with intracellular microbes, mainly by generating the formation of NO to enforce anti-microbial host responses, but also via the synthesis of polyamines, resulting in a pathogen-friendly nutritional environment [9,44,45,46]. A central enzyme controlling L-arginine levels in infected macrophages is ARG1, which cleaves L-arginine into ornithine and urea and thereby limits the availability of L-arginine for NO formation. Accordingly, the induction of ARG1 has been linked to an increase in tissue pathogen loads in the case of infections with *Leishmania*, *Trypanosoma* or *Mycobacteria* spp., whereas the inhibition or deletion of ARG1 led to improved infection control by the host [10,13,14,15,16,47]. So far, only little information has been available on the regulation of ARG1 upon infection with the intracellular bacterium *S*.tm and on a potential impact of ARG1 on the control of *S*.tm infection. A previous study suggested that ARG1 plays an important role in combatting *S*.tm infections in the murine macrophage cell line RAW264.7 and in mice by promoting NO production [48]. However, in the work presented here we did not find any effect of ARG1 on the course of infection using a model of acute systemic salmonellosis. Although *Arg1* was induced by *Salmonella* infection in BMDM in vitro and in spleens in vivo, genetic or pharmacological inhibition of ARG1 neither resulted in changes in *Salmonella* counts after 72 h of infection in various tissue compartments nor in alterations of the expression of several cytokines (*Il-6*, *Il-10* or *Tnf)* regulating anti-microbial effector pathways. Moreover, we did not observe an effect of ARG1 deletion on *iNos* expression and NO formation in vivo.

For studying the effect of ARG1 on the control of infections with *Salmonella,* we used the Tie2Cre^+/−^ARG1^fl/fl^ mouse model [13]. In vivo experiments with these mice have previously shown that the deletion of ARG1 in hematopoietic and endothelial cells improved the outcome of infections with *Leishmania major*, *Mycobacterium tuberculosis* and *Trypanosoma brucei* [10,13,14], whereas in our model of murine salmonellosis no reduction in the pathogen load was detected in liver, spleen and blood. We confirmed our findings obtained with the Tie2Cre mouse model by performing infection experiments, in which we blocked ARG1 using the orally bioavailable inhibitor CB-1158, which yielded similar results.

Compared to other in vivo studies, we used mice on a C57BL/6N background and not BALB/c mice. Infections of C57BL/6N mice tend to yield type 1 T-helper cell (Th1) responses, whereas BALB/c mice are more prone to develop Th2 responses. Upon activation of Th2 cells, more IL-4 is produced and therefore higher levels of ARG1 can be expected [49].

Lahiri et al., showed that the blockade of ARG1 with a different inhibitor, N^ω^-hydroxy-nor-L-arginine (nor-NOHA), in BALB/c mice led to reduced numbers of *S*.tm in the analysed organs. A further difference between their and our approach is the route of infection, as Lahiri and co-workers inoculated mice orally with the 12023 *S*.tm strain for 5 days, whereas we used a 72 h septicaemia model [13]. Additionally, the preparation of *S*.tm varied between our groups. Whereas Lahiri et al. diluted an overnight culture of *S*.tm to an OD600 of 0.2, we took some bacteria from the overnight culture and let them grow in fresh LB media to an OD600 of 0.5 in order to make sure that the *Salmonella* were in the logarithmic growth phase. An oral infection with *Salmonella* is obviously different from a systemic septicaemia induced by intraperitoneal injection of bacteria, both in terms of the severity of the elicited disease and with regard to the involved organs and immune responses [50,51,52,53].

Our results in murine systemic salmonellosis necessitate a comparison with previous data on the role of ARG1 during infections with other intramacrophage pathogens such as the protozoan parasite *Leishmania (L.) major*.

Paduch et al. infected Tie2Cre^+/−^ARG1^fl/fl^ C57BL/6N mice with *L. major* and found that the resolution of *L. major* infection was completely independent of ARG1, similar to our observation in the *S*.tm model. As the parasite itself did not induce upregulation of ARG1 and the immune response was dominated by a Th1 cytokine milieu, only low levels of ARG1 could be detected in the infected skin and draining lymph node [16].

In contrast, in *L. major* infections of BALB/c mice, the deletion of ARG1 was associated with a significant reduction in the tissue pathogen load from day 20 of infection onwards [10], while at earlier time points of infection the parasite burden was comparable in Tie2Cre^+/−^ARG1^fl/fl^ and Tie2Cre^−/−^ARG1^fl/fl^ BALB/c mice. In contrast to our *Salmonella* infection model, induction of *Arg1* in *L. major* infection in BALB/c mice was driven by a strong Th2 response releasing IL-4 and IL-13 and not by the pathogen itself. This explains why the ARG1-dependent effect in cutaneous leishmaniasis was only observed at later time points of the infection. The IL-4/IL-13-induced upregulation of *Arg1* was shown to restrict iNOS-derived NO production in the tissue. This is due to the fact that (a) both enzymes were expressed in the very same cell and that (b) *Arg1* and *iNos* were induced to a comparable level [10]. In *Salmonella*-infected macrophages in vitro and in *Salmonella*-infected C57BL/6N mice in vivo, however, the induction of *iNos* was much higher compared to that of *Arg1*. Thus, ARG1 could not really compete for the common substrate L-arginine, as was observed in the *L.* major BALB/c model.

In addition, previous evidence suggested that the NO pathway is primarily of importance for the control of prolonged infection of macrophages with *S*.tm, whereas the immediate early killing of the bacteria is mainly due to reactive oxygen species [8,54]. Therefore, alterations of NO formation may have only a limited impact on bacterial numbers after 72 h of infection. A limitation of our study is that due to ethical restrictions, we were not able to perform a survival experiment with wild-type and ARG1-deficient mice. To exclude that the unaltered bacterial burden in ARG1-gene-deficient or pharmacologically blocked mice is due to a non-functional NRAMP1, we investigated in vitro whether the presence or absence of functional NRAMP1 has an impact on the role of ARG1 during infection of macrophages with *S*.tm. As previously seen [23], macrophages with a functional NRAMP1 showed higher *iNos* activity. Therefore, they were superior in killing *S*.tm in vitro. However, we found that the expression of *Arg1* did affect the protective role of NRAMP1 in *S*.tm infection, and the pharmacological inhibition of *Arg1* did not alter the control of bacteria in C57BL/6N BMDM, irrespective of the NRAMP1 genotype.

Finally, it may well be that short-term induction or inhibition of ARG1 is not sufficient to critically reduce or enhance L-arginine levels in a way to influence the formation of NO, as we did not detect differences in nitrite levels with different ARG1 targeting strategies in infected mice [55]. In addition, we cannot exclude the possibility that the genetic deletion or pharmacological blockade of the ARG1 pathway is compensated by an increased expression of arginase 2 or metabolic reprogramming of other enzymes in the urea cycle, as the tissue urea levels were only marginally reduced upon treatment of mice with the ARG1 inhibitor CB-1158 [17,55,56,57].

In summary, we found a prominent induction of ARG1 in macrophages upon *Salmonella* infection and an opposite regulation of ARG1 by IL-4 and IFNγ. In contrast to the role of ARG1 for the control of infections with other intramacrophage pathogens, deletion or inhibition of ARG1 did not influence the control of systemic *S*.tm infection in mice. Based on our results, we conclude that targeting of ARG1 is not a treatment option in patients with systemic salmonellosis, including typhoid fever.

## Figures and Tables

**Table 1:** Sequences of primers and TaqMan probes.

Murine Gene	Forward Primer 5′-3′	Reverse Primer 5′-3′	Probe
*Hprt*	GACCGGTCCCGTCATGC	TCATAACCTGGTTCATCATCGC	ACCCGCAGTCCCAGCGTCGTC
*Arg1*	AACACGGCAGTGGCTTTAAC	GAGGAGAAGGCGTTTGCTTA	TGGCTTATGGTTACCCTCCCGTTG
*iNos*	CAGCTGGGCTGTACAAACCTT	CATTGGAAGTGAAGCGTTTCG	CGG GCA GCC TGT GAG ACC TTT GA
*Tnf*	TTCTATGGCCCAGACCCTCA	TTGCTACGACGTGGGCTACA	CTCAGATCATCTTCTCAAAATTCGAGTGACAAGC
*Il-6*	TGTTCTCTGGGAAATCGTGGA	AAGTGCATCATCGTTGTTCATACA	ATGAGAAAAGAGTTGTGCAATGGCAATTCTG
*Il-10*	CCAGAGCCACATGCTCCTAGA	TGGTCCTTTGTTTGAAAGAAAGTCT	TGCGGACTGCCTTCAGCCAGG

**Table 2:** Gene expression assays used for analysis of M1 and M2 marker genes.

Murine Gene	TaqMan® Genexpressionassays
*Hprt*	Mm00446968_m1
*Mrc1*	Mm00485148_m1
*Chil3*	Mm00657889_mH
*Il-6*	Mm00446190_m1
*Tnf*	Mm00443258_m1

**Table 3:** Antibodies used for immunoblotting.

Detected Protein	Antibody Dilution	Commercial Source	Catalogue Number
ARG1	1:2000	Novusbio (Littelton, CO, USA)	NBP1-32731
iNOS	1:1000	Abcam	ab3523
β-ACTIN	1:500	Sigma-Aldrich	A 2066

**Table 4:** Antibodies used for flow cytometry.

Antibody against	Conjugated Fluorochrome	Commercial Source	Catalogue Number
CD3	PE-eFluor 610	Invitrogen	61-0031-82
CD19	PE-eFluor 610	Invitrogen	61-193-82
CD49b	PE-CF594	BD Horizon	562453
CD11b	BB515	BD Horizon	564454
CD45	APC-R700	BD Horizon	565478
F4/80	BV421	BD Horizon	565411
Ly6C	BV510	BD Horizon	563040
Ly6G	Percp-eflour 710	Invitrogen	46-9668-82
iNOS	PeCy7	Invitrogen	25-5920-80

## Data Availability

All data presented within this study is available within the manuscript and the Supplementary Materials.

## Supplementary Materials

The following are available online at https://www.mdpi.com/article/10.3390/cells10071823/s1, Figure S1: Regulation of M2 and M1 markers in macrophages, Figure S2: Gating strategy of in vivo FACS staining.
