# Supplementary material for: Cytokine-Mediated Regulation of ARG1 in Macrophages and Its Impact on the Control of Salmonella enterica Serovar Typhimurium Infection
Source: Cells. 2021 Jul 19;10(7):1823. doi: 10.3390/cells10071823 (PMC8307077; doi:10.3390/cells10071823)
Supplement: Supplementary file 1 [file cells-10-01823-s001.zip › cells-1226086-supplementary.pdf]

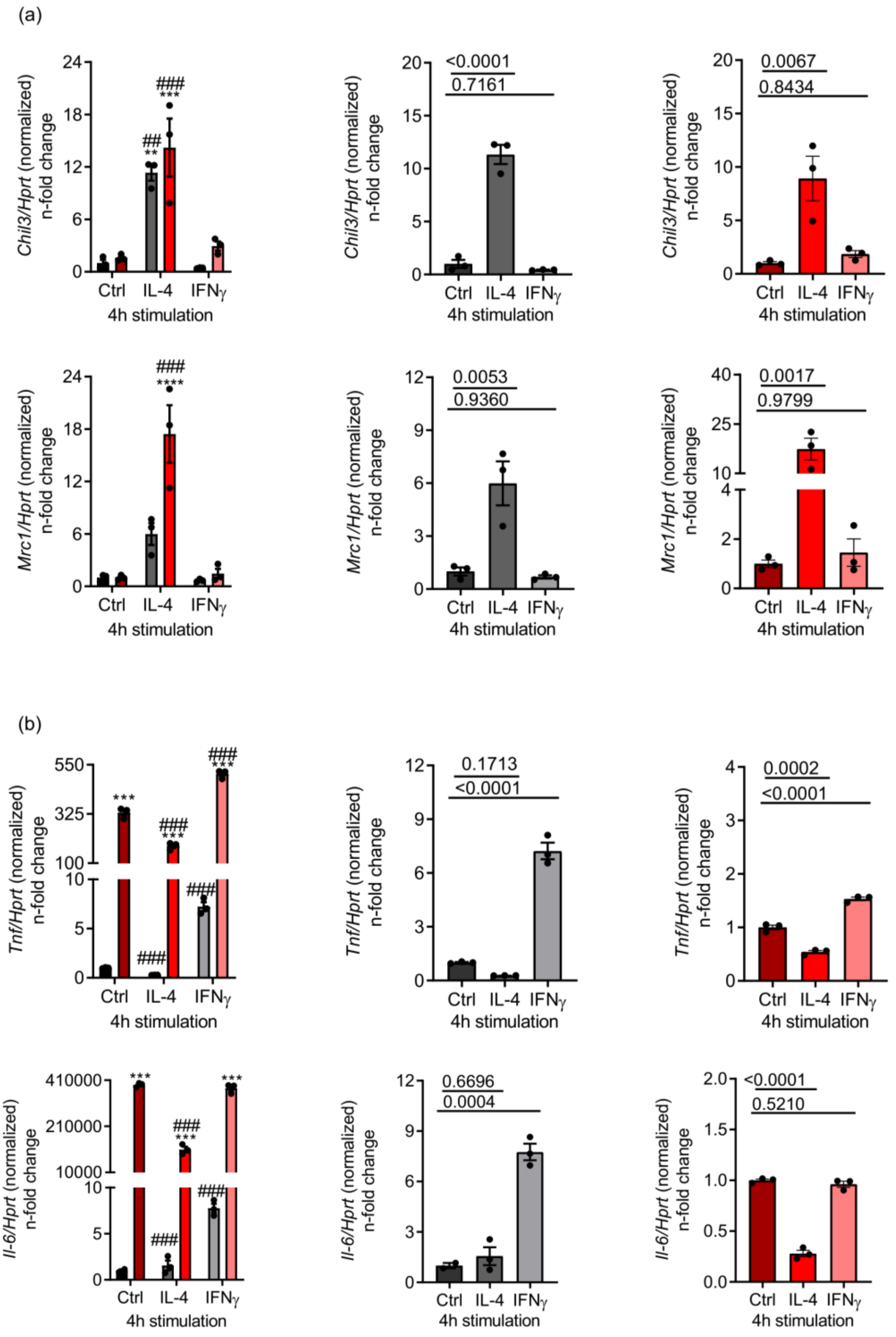

Figure S1: Regulation of M2 and M1 markers in macrophages. BMDM of C57BL/6N mice were uninfected (grey) and stimulated or infected (red) with *S*.tm and stimulated with IL-4 (10 ng/mL) or IFNγ (100 ng/mL) for 4 h. Uninfected and unstimulated BMDM were used as a control. (a) Regulation of *Chil3* and *Mrc1* expression due to infection and stimulation determined by RT-qPCR analysis. mRNA levels were normalized to the control *Hprt*. Ctrl samples were set to 1. (b) Regulation of *Tnf* and *Il-6* expression due to infection and stimulation determined by RT-qPCR analysis. mRNA levels were normalized to the control *Hprt*. Ctrl samples were set to 1. Significant differences as determined by two-way ANOVA are marked **;##=*p*-value <0.01; ***;###=*p*-value <0.001. * indicates significant differences to uninfected Ctrl; # depicts significant differences to infected Ctrl. Otherwise, three groups were statistically analysed by one-way ANOVA. Exact p-values are indicated.

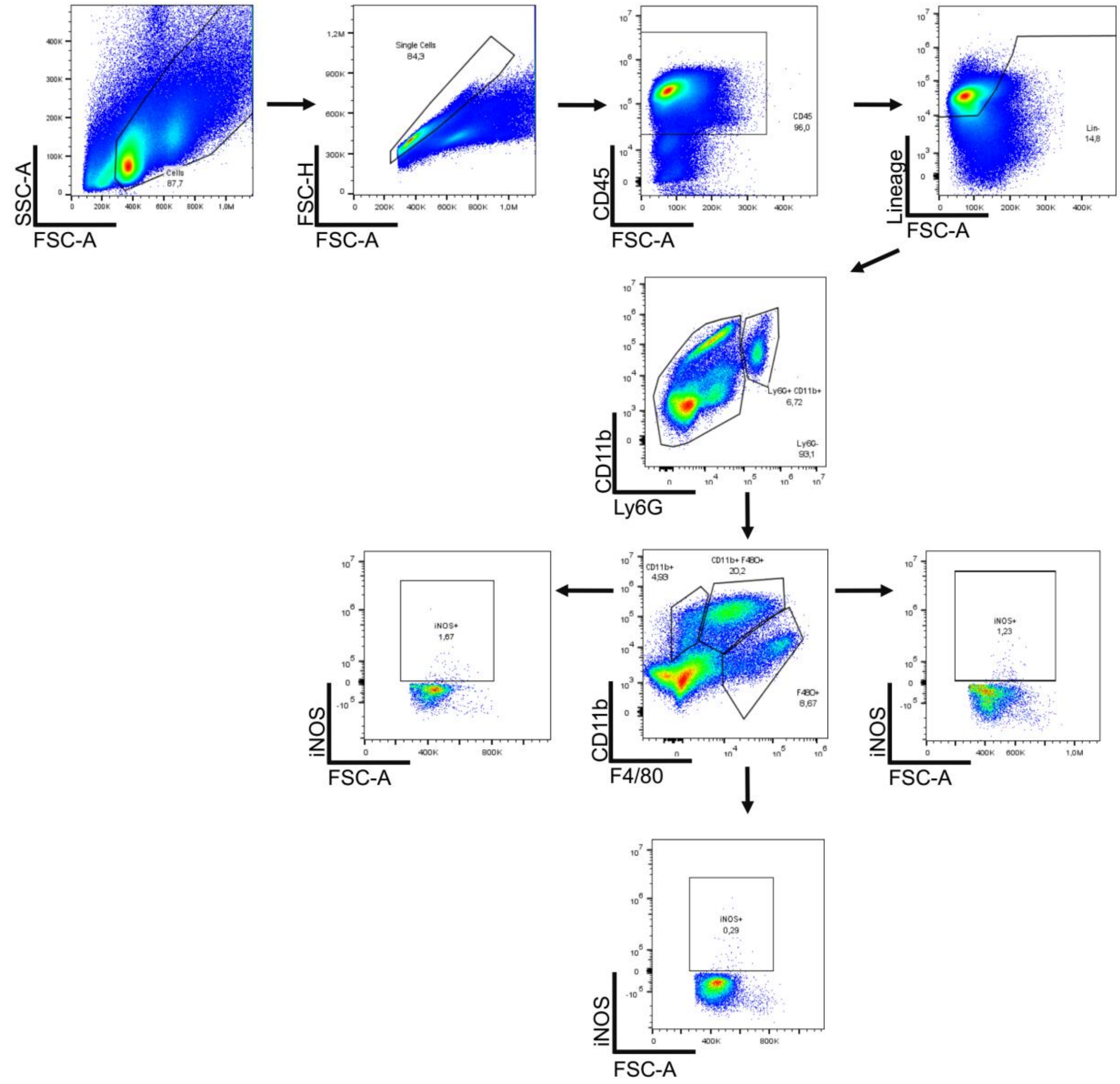

Figure S2: Gating strategy of *in vivo* FACS staining. Splenocytes of uninfected and infected mice were stained with various surface markers and intracellular staining against iNOS. Singlets were determined and only CD45+ cells were analysed. CD3, CD19 and CD49 (lineage) positive cells were excluded and Ly6G- cells were analysed for CD11b+, F4/80+ and CD11b/F4/80 double positive macrophage populations. iNOS was analysed in macrophage populations.
